# Primer-based cloning strategies for expression plasmid modification

**DOI:** 10.1016/j.mex.2026.104227

**Published:** 2026-10-23

**Authors:** Florian Weiss, Andrea K. Hönikl, Anna-Maria Hatzl, Vanessa Holzschuster, Anton Glieder, Lukas Rieder

**Affiliations:** a Christian Doppler Laboratory for Innovative Pichia pastoris Host and Vector Systems, Institute of Molecular Biotechnology, Graz University of Technology, 8010, Graz, Austria; b Institute of Molecular Biotechnology, Graz University of Technology, Petersgasse 14, Graz, 8010, Austria

**Keywords:** Molecular cloning, Expression plasmid modifications, Recombinant protein production, Mutants

## Abstract

The modification of expression plasmids, for example, to introduce mutations into an enzyme, is a cornerstone of modern biotechnology. Therefore, access to robust and straightforward techniques that enable i) introducing site-directed mutations, ii) exchanging smaller DNA regions, and iii) performing gene truncation in expression plasmids is highly valuable.

In this method, we present three versatile approaches that use standard laboratory equipment to achieve these modifications efficiently. The described procedures are refined adaptations of annealed oligo cloning and are particularly useful for scientists who only occasionally perform molecular biology work.

## Graphical abstract

**Figure:**
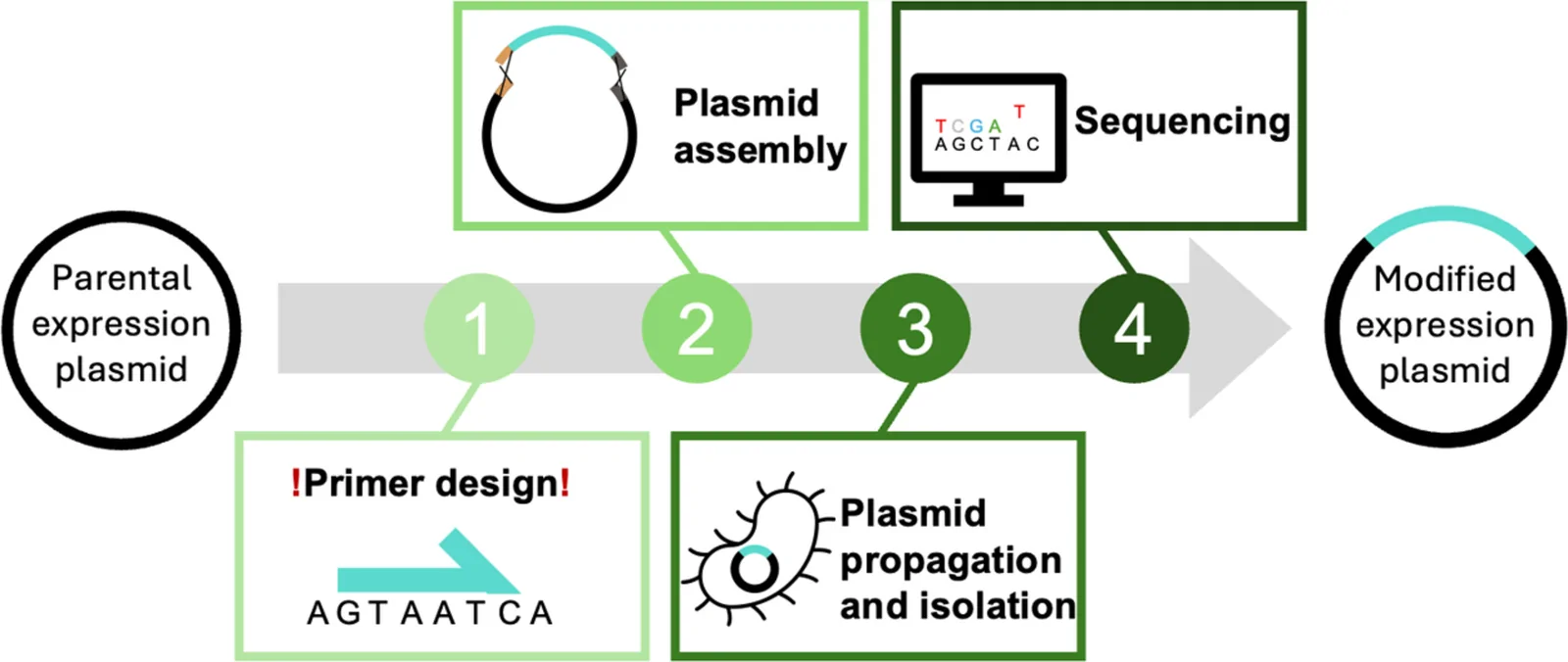

**Specifications table**

**Table:** 

**Subject area**	Biochemistry, Genetics and Molecular Biology
**More specific subject area**	DNA Cloning
**Name of your method**	Primer-Based Cloning Strategies for Expression Plasmid Modification
**Name and reference of original method**	Not applicable
**Resource availability**	• *In silico* cloning program (e.g. SnapGene; **NOTE:** a full license may be required, as the free version does not support the editing functions needed for the described procedures) • Standard equipment for PCR including high fidelity polymerase (e.g. Phusion DNA Polymerase; Thermo Fischer F530S) • Standard equipment for DNA electrophoresis • Gibson Assembly Cloning Kit (e.g. NEB E2611S) • Microcentrifuge with rotor for 1.5 ml reaction tubes (e.g. Eppendorf Centrifuge 5425/5425 R) • Shaking incubator (e.g. Eppendorf ThermoMixer® C) • Plasmid isolation Kit (e.g. Promega Wizard® Plus SV Minipreps DNA Purification Systems) • Gel-purification Kit (e.g. Promega Wizard® SV Gel and PCR Clean-Up System) • LB-Agar plates with appropriate selection agent (plasmid dependent) • NanoDrop • Sequencing provider (e.g. Microsynth AG)

## Background

Plasmids, i.e., self-replicating double-stranded DNA elements, are central to the genetic manipulation of organisms. In biotechnology, they are commonly used as vectors to introduce recombinant DNA into target organisms, thereby forming the basis for recombinant protein production in both academic and industrial settings.

In many applications, the DNA encoded on the plasmid needs to be modified to test, e.g., the impact of mutations on enzyme or promoter activity, or alternative signal peptides for efficient secretion of a target protein. Consequently, straightforward and robust cloning methods for introducing these modifications are highly valuable.

Here, we describe three strategies routinely used in our laboratory to i) introduce site-directed mutations, ii) exchange smaller DNA regions, and iii) perform gene truncation in expression plasmids. The strategies are built on the annealed oligo cloning (https://www.addgene.org/protocols/annealed-oligo-cloning/), a concept familiar to many working regularly in molecular biology.

A key feature of the presented method is the unconventional use of single-stranded DNA oligonucleotides (primers), which serve not only in PCR but are also directly integrated into the cloning process. This enables a streamlined workflow that reduces the number of cloning steps and minimizes reliance on additional reagents or specialized equipment.

All approaches rely on PCR and Gibson assembly [1,2] and can be implemented in any laboratory with basic equipment for molecular biology. To facilitate the successful application of the strategies, we provide detailed guidance on the most critical step, which is primer design. In addition, the method contains instructions for the subsequent *Escherichia coli* transformation procedure and plasmid isolation and sequencing.

Although we typically apply these strategies to modify *Komagataella phaffii* expression plasmids, the presented approaches are broadly applicable to virtually any plasmid. The simplified design and reduced experimental complexity make the presented method particularly useful for researchers who only occasionally perform molecular biology experiments, as well as for laboratories aiming to reduce reagent consumption, equipment requirements, and overall cloning costs.

## Method details

The presented method relies on two major steps: i) primer design and ii) plasmid assembly, including sequence verification. Whereas the plasmid assembly and sequencing follow established protocols using commercially available reagents and service providers, the correct primer design marks the critical step for the efficient modification of expression plasmids. Hence, the main part of the method will focus on the design of the correct primers.

## Primer design for plasmid linearization and assembly

To be able to provide detailed guidelines on correct primer design for the successful application of the method, the primer design for our top three applications, i.e., i) introducing site-directed mutations; ii) exchanging ∼60 bp DNA regions; iii) truncation of gene elements, is outlined below.

### Application 1: introducing point mutations

This application is useful for site-directed and rational mutagenesis in already existing expression plasmids. The technique comprises two steps. In the first step, the expression plasmid is linearized by PCR at the anticipated position. In the second step, Gibson assembly is used for the site-directed insertion of the primer containing the mutations (Fig. 1).

**Fig. 1:**
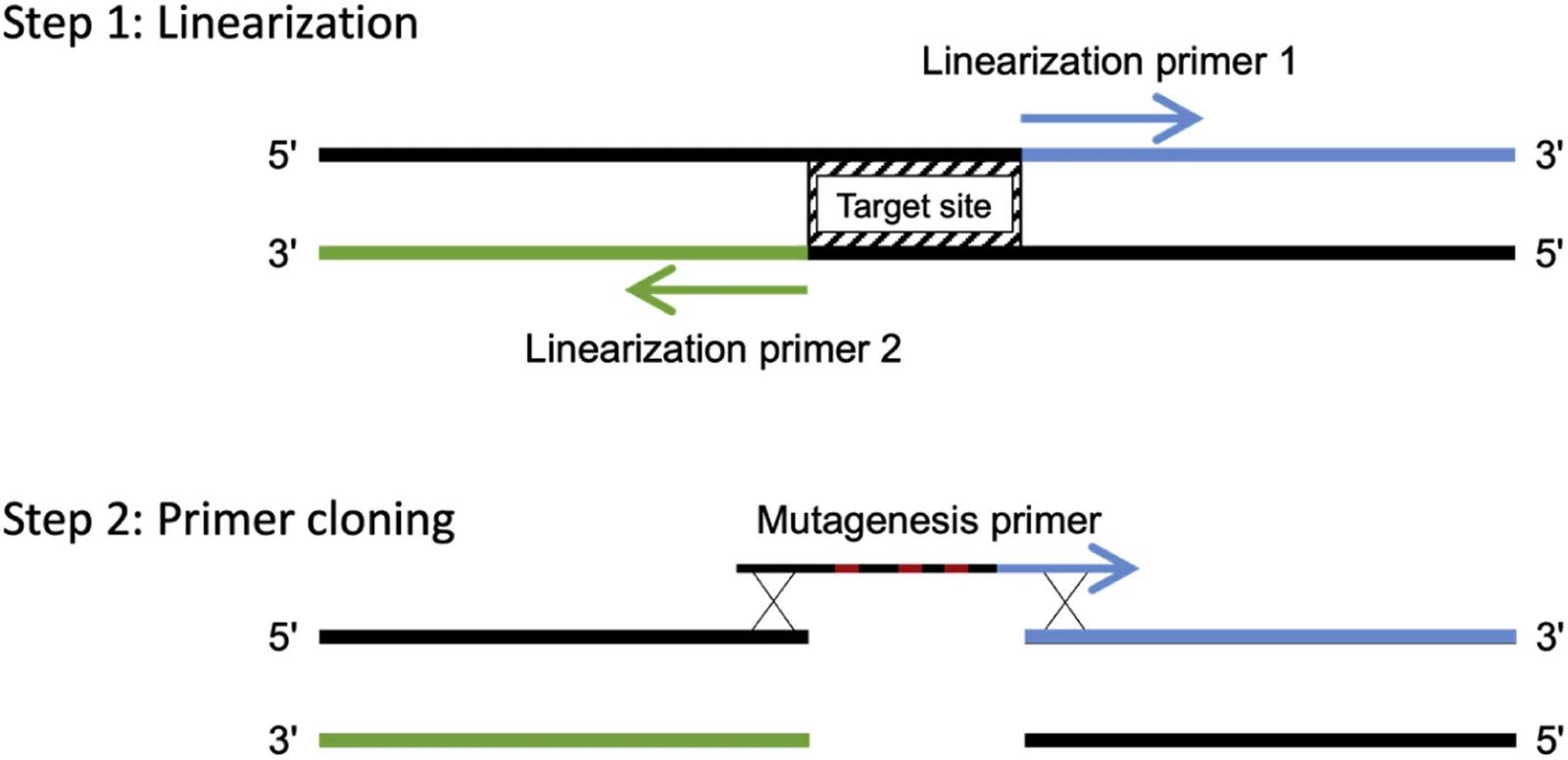
Schematic illustration of the 2-step protocol to perform site-directed, rational mutagenesis using primer-based cloning approaches. Step 1: plasmid linearization by PCR. Step 2: Site-directed insertion of mutagenesis primer using Gibson cloning.

The mutagenesis primer is added to the Gibson assembly reaction as a single-stranded DNA fragment and anneals to the complementary regions of the linearized plasmid backbone. The polymerase activity of the assembly mixture subsequently fills the remaining gap, thereby generating the complementary strand, while the DNA ligase seals residual nicks to yield a double-stranded circular plasmid.

a. Open the plasmid map of the expression plasmid containing the to-be-modified DNA sequence in SnapGene. **NOTE:** We generally recommend having one plasmid map for each plasmid that is made in the laboratory. This allows keeping track of the plasmid history and makes it easier to further build on already existing plasmids.
b. Select the DNA region downstream of the target site to design “Linearization primer 1”. **NOTE:** It is possible to linearize the plasmid using restriction enzymes [3]. However, in our experience, this results in a higher background of cells with the unmodified plasmid due to incomplete digestion and requires that a suitable restriction site is available.

Select a DNA region with a melting temperature (Tm) of ∼58 °C that is ≥18 bp. Ensure a “G” or “C” on the 3′-end (Fig. 2). SnapGene will automatically calculate the Tm once at least 8 bp are selected. **NOTE:** We routinely design all primers meant for PCR to a Tm of ∼58 °C, as this allows performing PCRs with different primers in the same thermocycler.

c. Press ctrl/cmd + R to open the primer design tool and select “Top Strand”.
d. Give the primer a unique name by typing in the “Primer” field on the top left (Fig. 3).

**Fig. 3:**
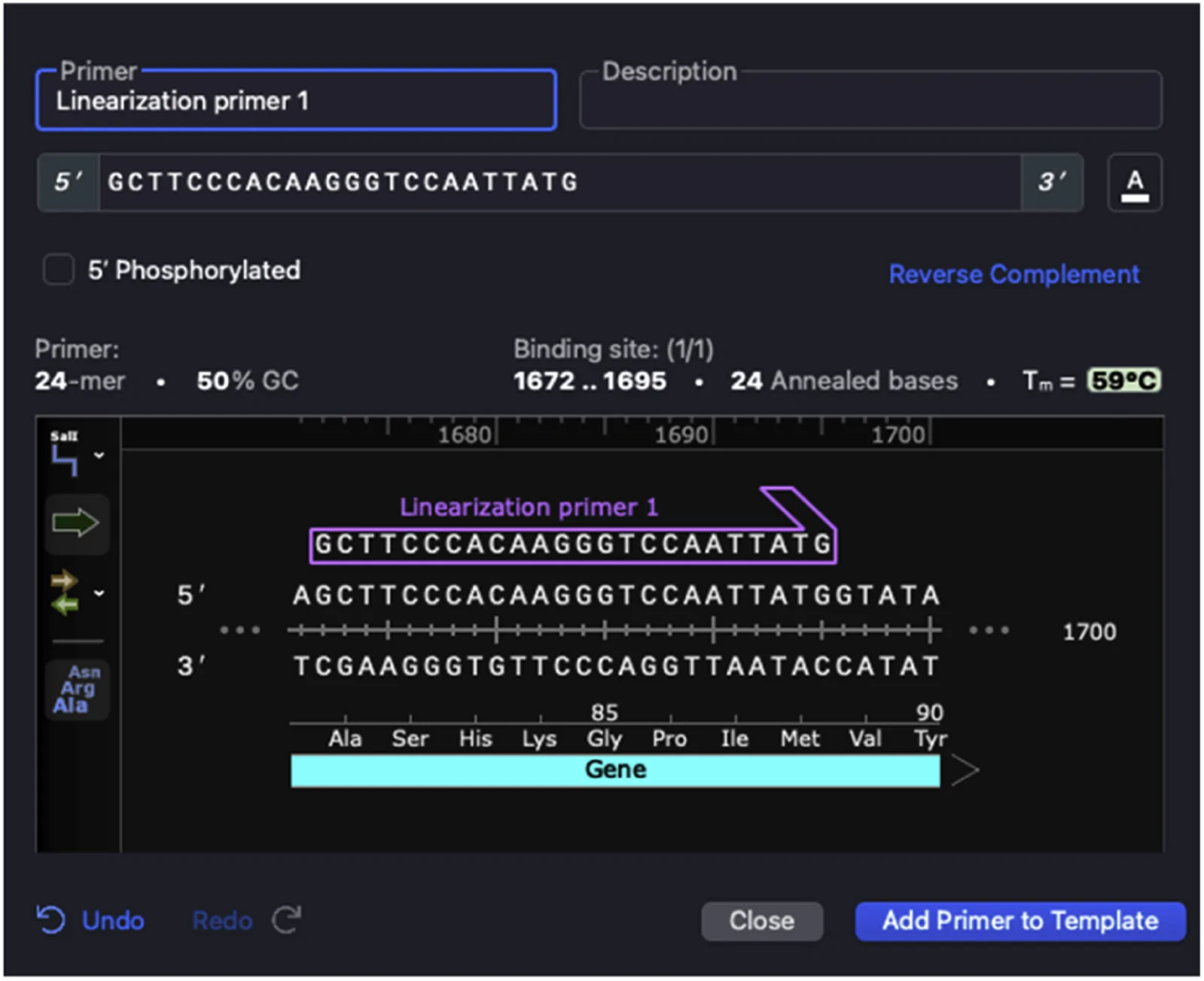
SnapGene screenshot showing the primer design tool. The newly designed primers are shown as purple arrows. The primer name can be adjusted in the box to the top left titled “Primer” (blue box).
e. Select “Add Primer to Template” to add the primer to your plasmid map.
f. Repeat the same procedure to design the “Linearization primer 2” upstream of the mutation site. Note that for designing the reverse primer, select “Bottom Strand” in the primer design tool. **NOTE:** The direction of the arrow framing the primer sequence indicates the amplification direction.
g. If done correctly, the plasmid map now contains two primers with similar Tm that flank the mutation site with arrowheads in opposite directions (Fig. 4). **NOTE**: Likely, it is not possible to design primers with identical Tm. But it is advisable to have Tm within a ∼2 °C range, which can be adjusted by altering the primer length.

**Fig. 4:**
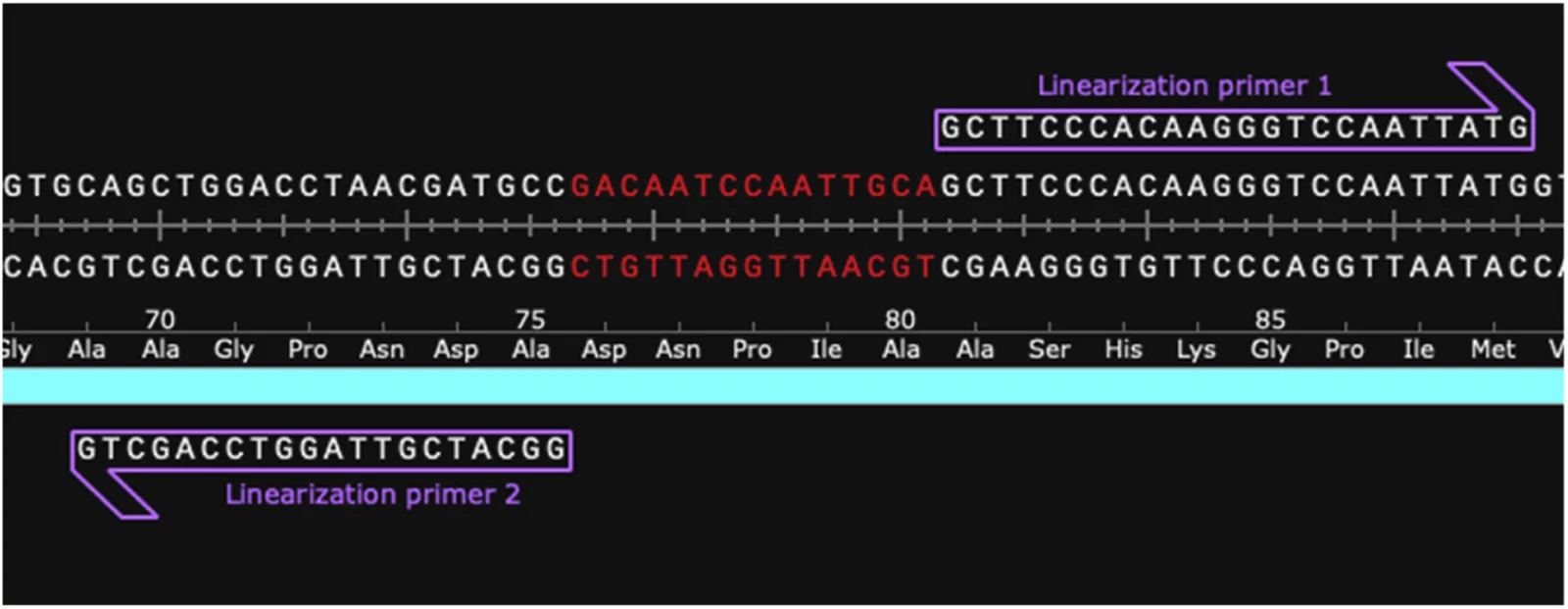
SnapGene screenshot showing linearization primer 1 and 2 (purple arrows) flanking the mutation site (red letters). Note the amplification direction of the primers indicated by the arrowhead.
h. To design the regions for Gibson cloning, select a DNA region upstream of the to-be-altered region with a Tm of ∼55 °C that will lie within the sequence of the “Linearization primer 2”.
i. Press ctrl/cmd + T to add a feature to the file. Name the feature “Cloning upstream”.
j. Repeat the same step for the region downstream of the mutation site and call the feature “Cloning downstream” (Fig. 5).

**Fig. 5:**
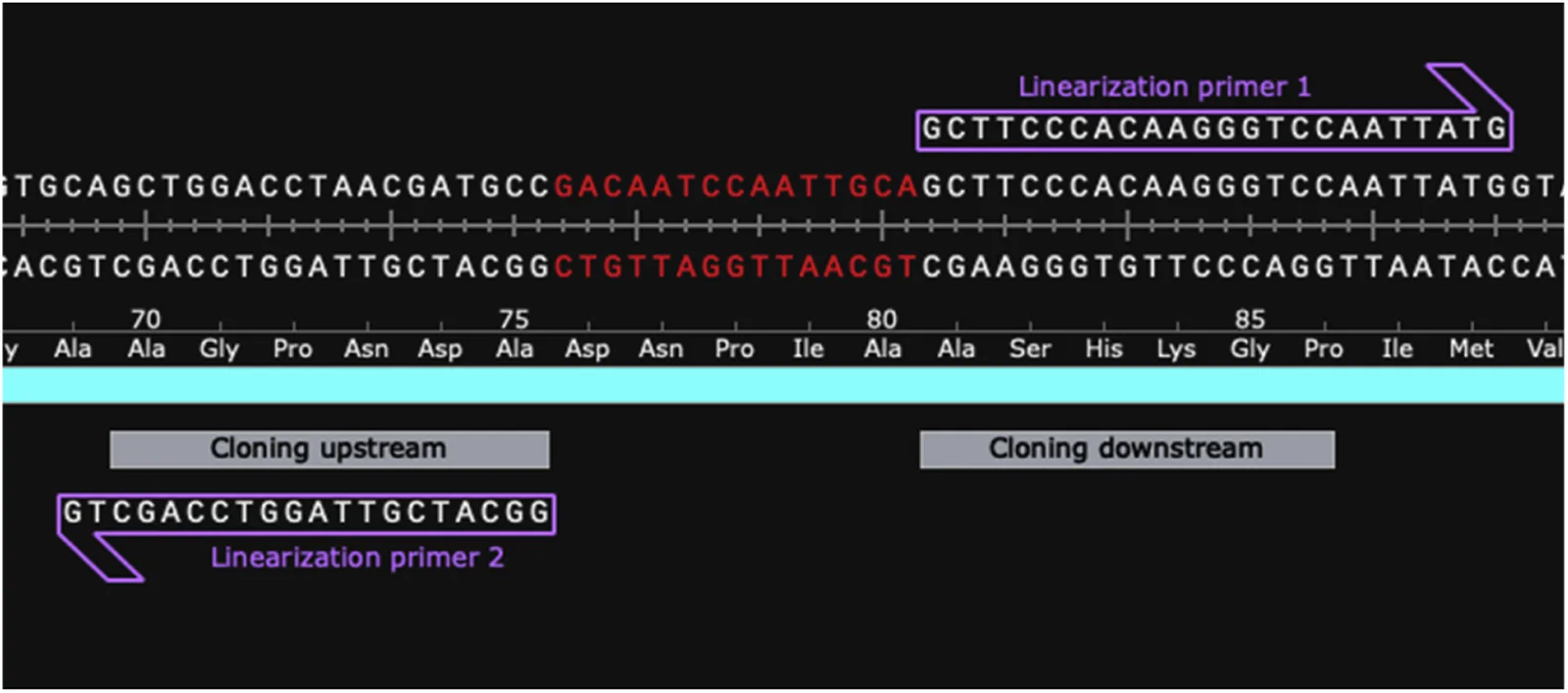
SnapGene screenshot showing linearization primer 1 and 2 (purple arrows) and the regions (grey bars) required for Gibson cloning flanking the mutation site (red letters).
k. Introduce the desired mutation(s) in the targeted region. **NOTE:** It is also possible to saturate one/ several positions by using this approach. For this, replace the codon(s) at the desired position(s) in the gene with “NNK” in the primer. When doing this, be aware that you need to select enough transformants to adequately cover the library; e.g., for one saturated position, at least 320 independent transformants should be screened, corresponding to a 10-fold oversampling of the 32 possible NNK codons. Alternatively, you can design separate cloning primers for each desired amino acid and assemble the respective plasmids individually.
I. Once homologous regions for Gibson cloning are defined and the mutations have been inserted, the mutagenesis primer can be designed. Select the whole sequence covering the features “Cloning upstream” and “Cloning downstream”, press ctrl/cmd + R and select “Top Strand”.
m. Give the mutagenesis primer an appropriate name and add the primer to the plasmid map.
n. Add as many mutation primers with distinct mutations as you want by repeating steps l to n.
o. Restore the original gene sequence (Fig. 6). The primers will now indicate the mutations introduced.

**Fig. 6:**
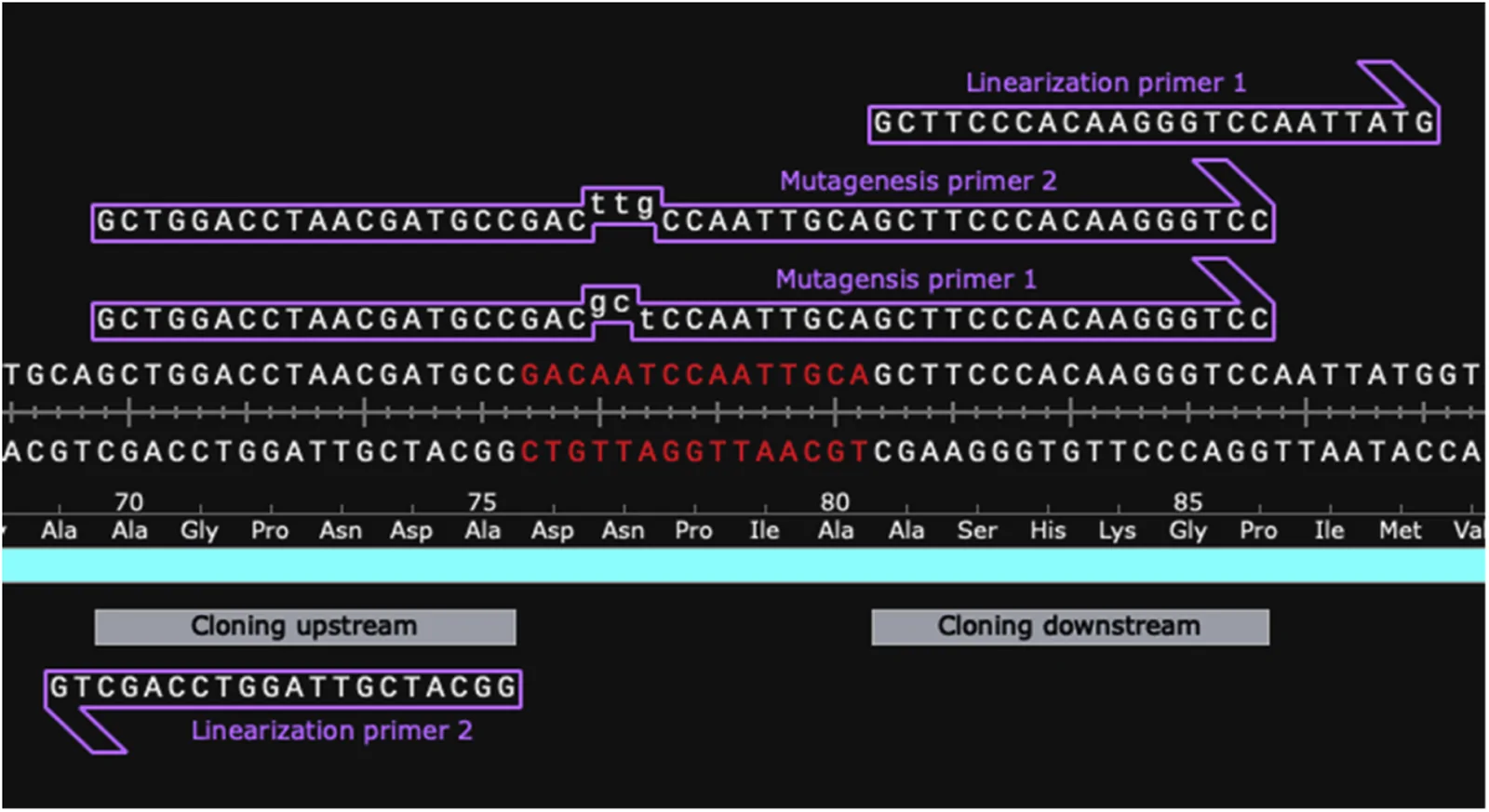
SnapGene screenshot showing the final design for introducing point mutations by purely primer-based cloning approaches. The longer purple arrows represent the mutagenesis primers, while the “bumps” in the primer sequences indicate the positions where nucleotides have been altered to introduce the desired mutations. NOTE: In theory, the length of the region that can be mutated by this method is the maximum primer length that can be synthesized minus the homologous regions needed for cloning. Still, at some point, primers become more expensive than gene fragments, making the approach economically unreasonable.
p. Order primers from your local provider.

**Fig. 2:**
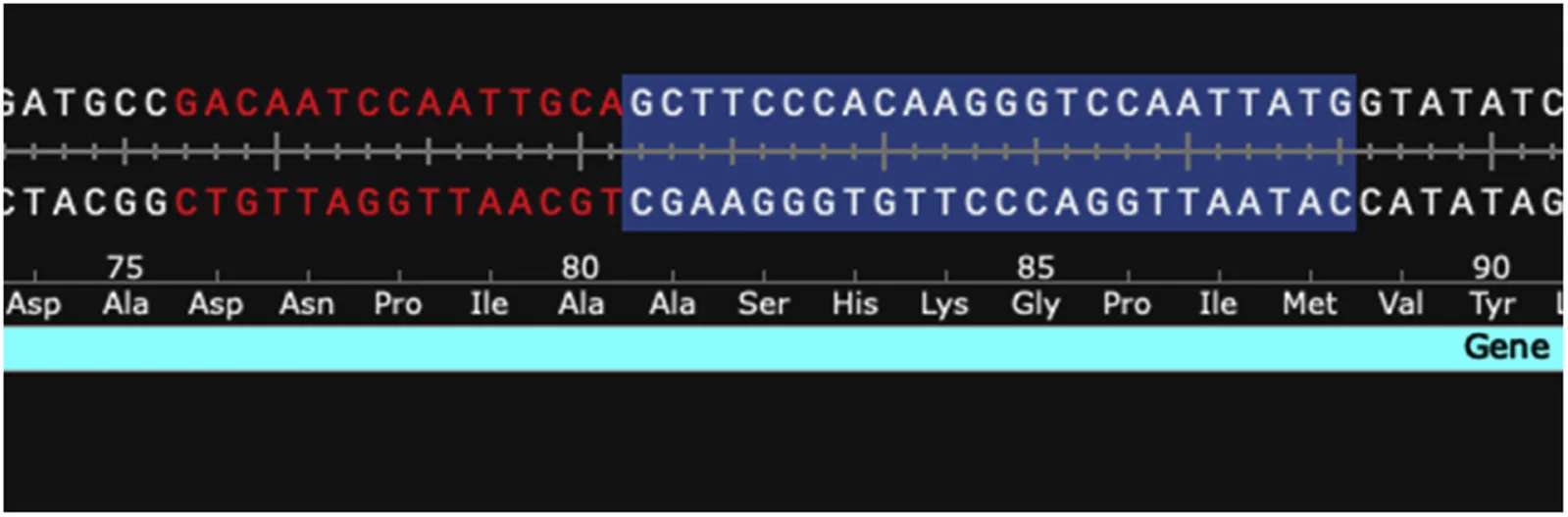
SnapGene screenshot showing the mutation site (red letters) and the sequence selected for “linearization primer 1” (highlighted in blue).

## Application 2: region replacement

This application is useful to exchange shorter DNA regions, like signal peptides or loop regions. Identical to application 1, two steps have to be performed. In this application, however, two primers are fused during cloning, which allows bridging regions of ∼70 bp using two 60 nt primers. As outlined in Application 1, a PCR to linearize the plasmid at a defined position is performed in the first step. In the second step, Gibson cloning is used to fuse the linearized plasmid and two primers (Fig. 7) to obtain a circular plasmid.

**Fig. 7:**
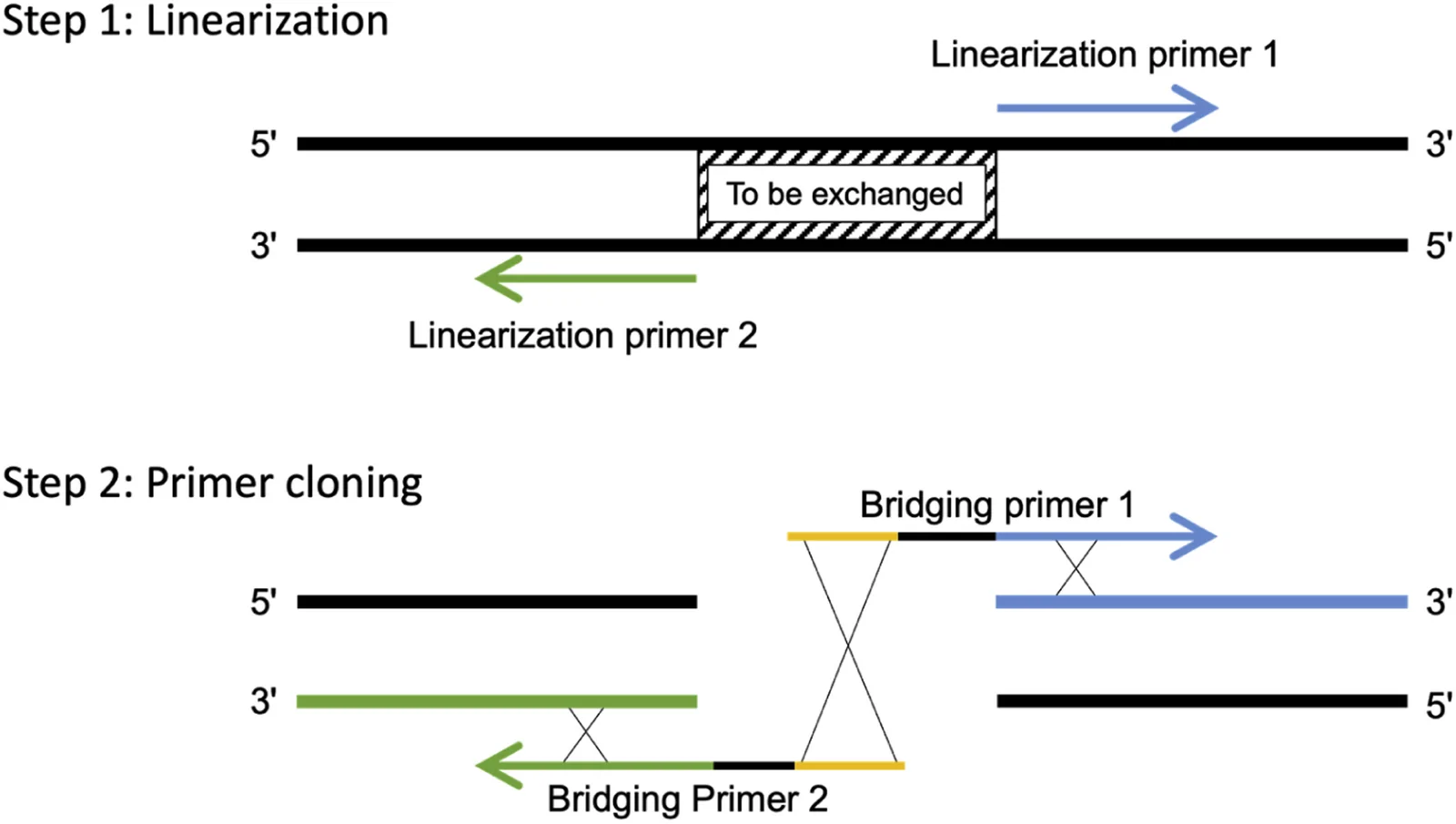
Schematic illustration of the 2-step protocol to exchange regions of up to 70 bp using two 60 nt bridging primers. Step 1: Linearization of the plasmid at a defined position by PCR. Step 2: Gibson cloning using the linearized plasmid and two bridging primers.

During Gibson assembly, the two single-stranded bridging primers anneal to the complementary regions of the linearized plasmid backbone and to each other through their designed overlap. The polymerase activity of the assembly mixture fills the remaining gaps to generate the complementary strands, while the ligase seals residual nicks, resulting in a double-stranded circular plasmid.

a. The linearization primers for linearizing the plasmid at a defined position are designed as outlined for Approach 1, steps a to k.
b. Adjust the DNA sequence between the two linearization primers as desired.
c. Start from the “Cloning downstream” feature and highlight a total of 60 bp towards the “Cloning upstream” feature. **NOTE:** As mentioned before, the length of the primers that can be used in this approach is variable.
d. Press ctrl/cmd + R and select “Top Strand”.
e. Give the primer a unique name and add it to the plasmid map.
f. Select a DNA sequence at the 5′ of the bridging primer 1 having a Tm of ∼55 °C. **NOTE:** The sequence length required to achieve a Tm of ∼55 °C is typically between 15 bp and 20 bp. In case a longer primer is needed to reach this Tm, the region that can be inserted is shorter.
g. Press ctrl/cmd + T to make a new feature called “Cloning overlap”.
h. Select the DNA sequence from “Cloning upstream” to “Cloning overlap”.
i. Press ctrl/cmd + R and select “Bottom Strand”.
j. Give the primer a unique name and add it to the template (Fig. 8).

**Fig. 8:**
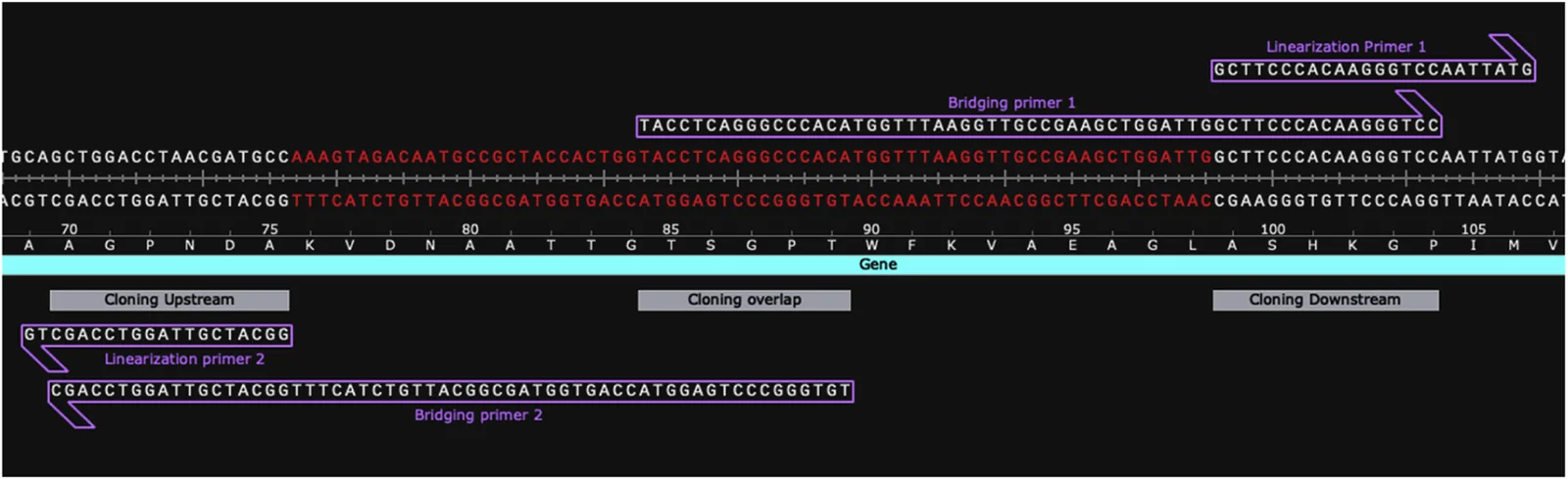
SnapGene screenshot showing the final *in silico* design for the exchange of a 70 bp DNA region (red letters) using two 60 nt primers. The longer purple arrows indicate bridging primers introducing the exchanged DNA during cloning.
k. Order primers from your local provider.

## Application 3: truncation

This application allows the truncation of genes, which is, e.g., useful to remove domains from the N- or C-terminus of an enzyme [4]. Like for the other applications, two steps are needed, but only one primer pair must be designed. This is because the amplification already introduced to homologous regions needed for cloning, which allows the PCR product to be directly added to the Gibson reaction, enabling DNA circularization. Hence, this approach has no limitations concerning the length of the truncation (Fig. 9).

a. Design the Linearization primer 1 and add the “Cloning downstream” feature as outlined in application 1, steps a to k. When working at the C-terminus of genes, make sure the stop codon is included at the 5′-end of “Linearization primer 1” (Fig. 10). Correspondingly, if you want to remove N-terminal portions, ensure that the start codon is included in the linearization primer.

**Fig. 10:**
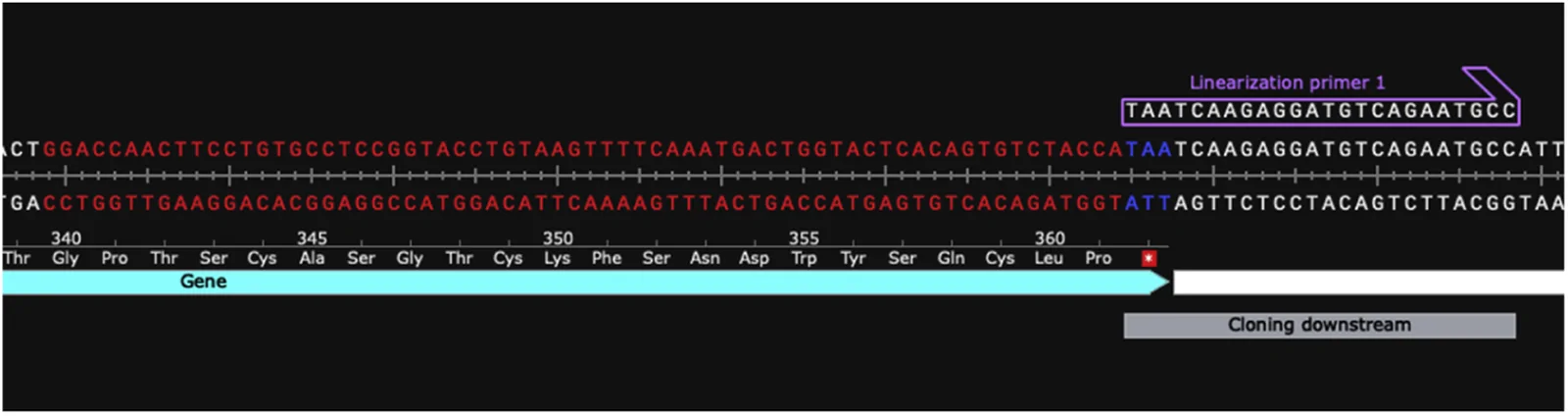
SnapGene screenshot showing linearization primer 1 and the homologous region needed for cloning downstream of the truncation site (red letters). Note that the stop codon (blue letters) is included on the 5′-end of the linearization primer.
b. Perform a virtual truncation by removing the relevant sequence from the plasmid map to simplify truncation primer design.
c. Mark a sequence upstream of the “Cloning downstream” feature having a Tm of 58 °C.
d. Select the sequence with a Tm of ∼58 °C and the “Cloning downstream” feature.
e. Press ctrl/cmd + R and select “Bottom Strand”. **NOTE:** The Tm is critical for the success of this strategy. The Tm of the primer region responsible for recircularization must be higher than 50 °C, as required for Gibson assembly.
f. Give the primer a unique name and add the “Deletion primer” to the template (Fig. 11).

**Fig. 11:**
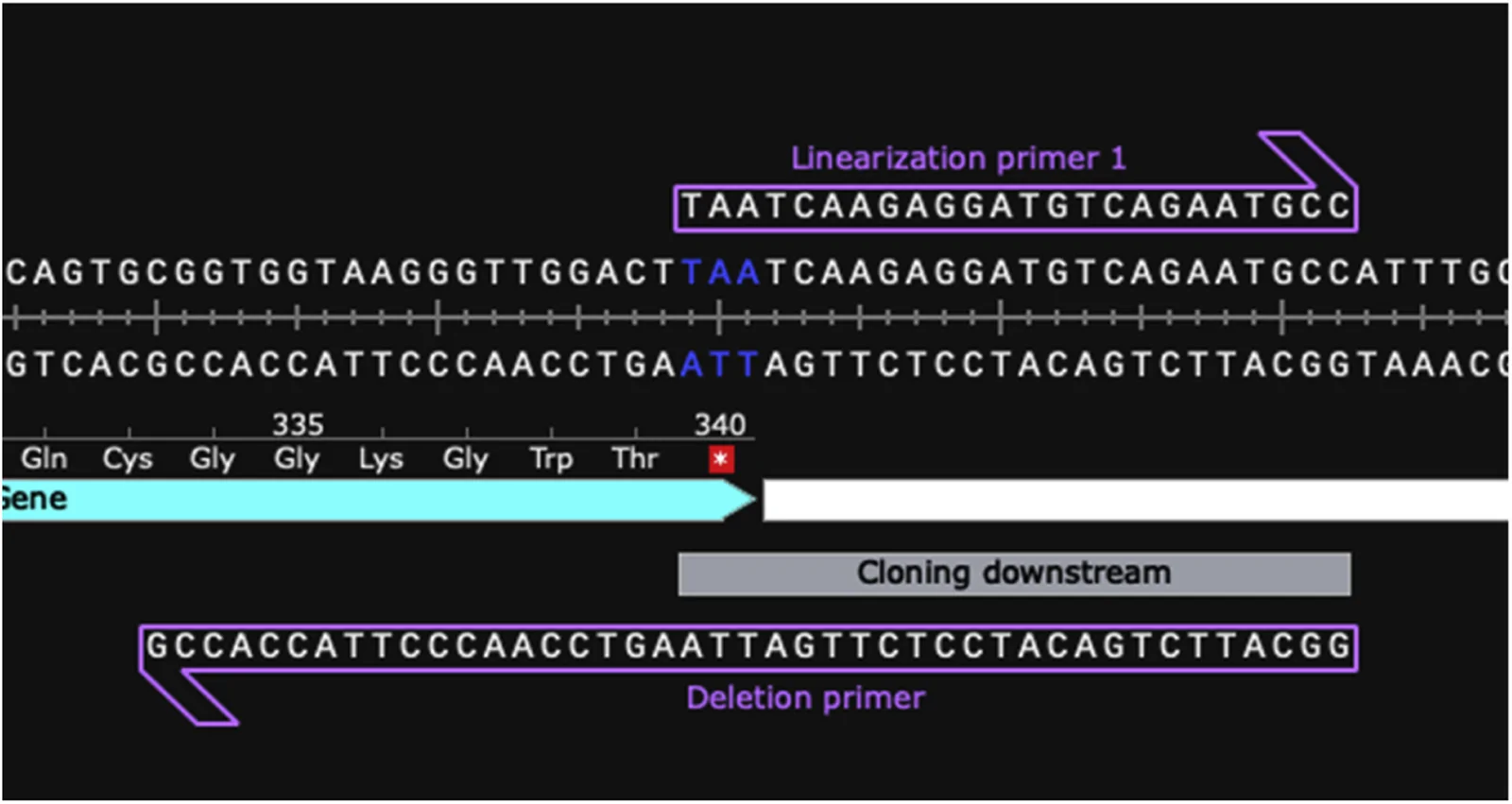
SnapGene screenshot showing the arrangement of primers for gene truncation. Please note the positioning of the stop codon (blue letters) at the 3′-end of the truncated gene.
g. Order primers from your local supplier.

**Fig. 9:**
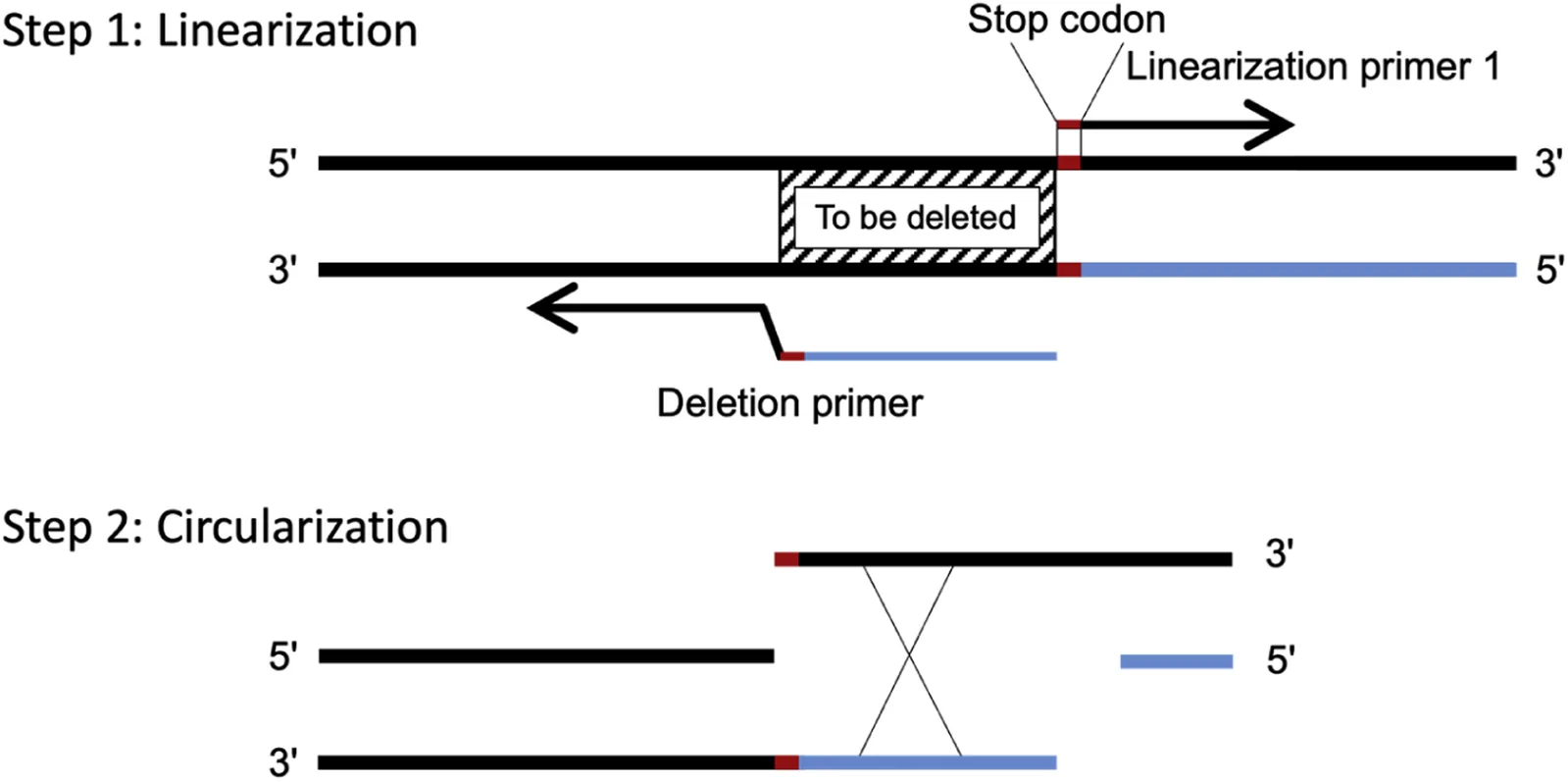
Schematic illustration of the two-step protocol for gene truncations.

## Plasmid assembly

Once the primers are obtained from the commercial vendor, the modified plasmids can be assembled. For this, the backbone, i.e., the non-modified part of the plasmids, is PCR amplified. Subsequently, Gibson assembly is performed in the presence (Application 1& 2) and absence (Application 3) of additional primers. Finally, the newly assembled plasmids are propagated and recovered from *E. coli*, and sequence verified before use.

### PCR for amplification of the plasmid backbone

a. Spin down the tubes containing the lyophilized primer DNA for 1 min at full speed using a bench-top centrifuge.
b. Dilute lyophilized primers to a concentration of 100 µM (100 pmol/µl) using ddH_2_O.
c. Thaw reagents needed for the PCR on ice.
d. Prepare the PCR according to Table 1 **NOTE:** This will be dependent on the polymerase used

**Table 1:** PCR Master Mix for 50 µl reaction.

Reagent	Amount
DNA template	X µl (0.2 ng total)
Phusion DNA polymerase	0.5 µl
Primer 1 (10 µM)	2.5 µl
Primer 2 (10 µM)	2.5 µl
5x HF Buffer 5x	10 µl
dNTPs (10 mM)	1 µl
ddH_2_O	33.5-X µl
e. Perform the PCR according to the cycling conditions outlined in Table 2. **NOTE:** If primers are designed to have identical melting temperatures, PCRs with similar amplicon lengths (±500 bp) can be performed simultaneously. Elongation time is adjusted to the longest fragment. Also, the exact conditions will be dependent on the polymerase used.

**Table 2:** PCR cycling profile for the amplification of the plasmid backbone.

Steps	Temperature	Time	Cycles
Initial Denaturation	98 °C	30 s	1
Denaturation	98 °C	10 s	30 cycles
Annealing	58 °C*	20 s	
Extension	72 °C	30 s/ kb	
Final extension	72 °C	10 min	1
Hold	4 °C	forever	1

*if primers are designed according to the outlined guidelines. Otherwise, adapt accordingly.
f. Prepare a 1.5% (w/v) agarose gel according to the needs of the DNA stain used. Make sure that the gel has sufficient wells (1 per PCR tube, plus 1 for a DNA ladder).

g. Spin down the PCR and mix 1 µl 6x Loading Dye with 5 µl of each of the PCR reactions and load the sample onto the agarose gel.
h. Perform an agarose gel electrophoresis using appropriate conditions (e.g., 120 V, 400 mA, 30 min)
i. If an amplicon with the correct length is observed, recover the DNA of the remaining 45 µl of the PCR reaction using the PCR clean-up system according to the manufacturer's protocol. **NOTE:** A *Dpn*I digestion of the PCR product can optionally be performed prior to purification to reduce background originating from the methylated parental plasmid template. In our workflow, only 0.2 ng of plasmid template is used per 50 µl PCR reaction (corresponding to a final template concentration of 4 pg/µl). This low template concentration is sufficient for efficient amplification while minimizing the amount of parental plasmid carried into subsequent steps. For this reason, we do not routinely perform *Dpn*I digestion, and it is therefore not included as a standard step in the presented protocol. However, if substantial background from the parental plasmid is observed, *Dpn*I digestion can be used to reduce this background. **NOTE:** We recommend increasing the reaction volume in case more DNA is required (e.g., for multiple cloning attempts) and pooling identical PCRs before purification to maximize the DNA recovery.
j. Assess DNA concentration using a NanoDrop or similar device. Typically, a DNA concentration of ∼200 ng/µl can be expected.
k. Dilute the PCR-amplified plasmid backbone to a concentration of 100 ng/µl using ddH_2_O.

### Gibson assembly, *E. coli* transformation and plasmid isolation

#### Gibson cloning

a. Spin down the tube containing the lyophilized mutagenesis/bridging primers at maximum speed for one minute.
b. Resuspend the primers to a concentration of 100 µM (100 pmol/µl) using ddH_2_O.
c. Prepare a 1:250 dilution of the primers using ddH_2_O in a fresh 1.5 ml reaction tube. The final concentration of each primer in the Gibson assembly reaction is 0.02 µM.
d. For each cloning reaction, thaw one 10 µl aliquot of the assembly mixture on ice.
e. Depending on the application, prepare the Gibson reaction on ice according to Table 3.

**Table 3:** Gibson assembly pipetting schema for the three distinct applications.

	Mutagenesis (Approach 1)		Region exchange (Approach 2)	Truncation (Approach 3)
Reagent	Amount (µl)			
Assembly master mix*	10	10		10 µl
Primer 1 (0.4 µM)	1	1		0
Primer 2 (0.4 µM)	0	1		0
Plasmid backbone (100 ng/µl)	1	1		1
ddH_2_O	8	7		9
Total	20	20		20

*Depending on the Gibson assembly mixture used.
f. Mix the solution by flicking the tubes, then perform a quick spin using a centrifuge.
g. Incubate the cloning mixtures at 50 °C for 60 min for plasmid assembly.

#### *E. coli* transformation

For the transformation of competent *E. coli* cells, we generally recommend following the manufacturer's instructions. Still, in case an instruction is missing, the following describes a standard protocol for the transformation of chemically competent *E. coli* cells.

a. Preheat the thermomixer to 42 °C.
b. Prepare two LB agar plates with the appropriate selection agent for each transformation.
c. Thaw one aliquot of competent *E. coli* cells per transformation on ice.
d. Perform a quick spin on the cloning mixtures from the previous step using a benchtop centrifuge.
e. Add 2.5 µl of the cloning mixture to one 50 µl aliquot of the competent cells. Keep the cells on ice while doing this.
f. Thoroughly mix cells and DNA by flicking the tubes.
g. Incubate the transformation reaction on ice for ∼5 min.
h. Place the transformation reaction on the thermomixer preheated to 42 °C.
i. After 30 seconds of incubation, add 500 µl of SOC medium and lower the temperature to 37 °C.
j. Regenerate the cells for at least 60 min at 37 °C.
k. Plate 100 µl of the transformation mixture on LB agar plates with the appropriate selection agent.
l. Spin down the remaining 400 µl of the transformation reaction for 2 min at 6000 g using the bench-top centrifuge.
m. Remove excess liquid and resuspend the *E. coli* cells in the remaining SOC medium
n. Plate the resuspended cells on LB agar plates with the appropriate selection agent.
o. Incubate the agar plates with the cells overnight at 37 °C.

#### Plasmid isolation

a. Take one fresh agar plate with the appropriate selection agent per successful transformation and divide it into quarters using a marker pen.
b. Streak out one transformant per quarter and incubate the plates at 37 °C overnight for biomass formation for subsequent plasmid isolation. **NOTE:** The streak out should be dense.
c. Perform plasmid isolation using the Wizard® Plus SV Minipreps DNA Purification Systems with small deviations from the manufacturer’s instructions. Instead of pelleting an overnight culture, add 250 µL of cell resuspension solution to a fresh 1.5 mL microcentrifuge tube. Then, using an inoculation loop, collect *E. coli* biomass from one quadrant of the LB agar plate and resuspend the cells in the solution. **NOTE:** Proper resuspension of the cells will typically increase the yield of plasmid DNA obtained.
d. Assess DNA concentration using the NanoDrop. Typically, the DNA concentration obtained should be in the range of 200 to 700 ng/µl, depending on the user’s experience and quality of materials used.

#### Sequencing

a. For preparing the sequencing reaction, follow the instructions of your local provider. Typically, one Sanger sequencing reaction requires ∼1000 ng of plasmid DNA and covers ∼1000 bp. Still, this may depend on the provider and the quality of your DNA preparation.
b. The sequencing primer should be chosen to bind at least 50–60 bp up-/ downstream of the mutation site to ensure reliable results.
c. Once finished, download your sequencing results.
d. Open the plasmid map corresponding to the sequenced plasmid in SnapGene.
e. Press ctrl/cmd + L to open the alignment tool in SnapGene.
f. Drag and drop the sequencing files, preferably containing chromatograms, into the box with the heading “Align with”.
g. If sequencing worked, SnapGene will automatically align the sequencing results to the reference sequence.
h. Click on “Map” in the bottom left corner to get an overview of your sequencing results. You will see arrows indicating the direction of the sequencing reaction relative to your reference. A full arrow indicates that the sequencing results match the reference. In case the arrow shows gaps, the sequencing results indicate mutations.
i. Click on “Sequence” in the bottom left corner to investigate the sequencing results in detail. **NOTE:** If gaps or mismatches are observed at the start or end of the sequencing results, this is most likely due to reduced sequencing accuracy at the start and end of the run. If uncertainty remains, resequencing the plasmid using primers binding ∼100 bp upstream of the region of interest is a typical solution.
j. Choose a plasmid preparation that has been sequence-verified as your new expression plasmid variant and label the tube accordingly.

## Method validation

The expected outcome of the presented method is sequence-verified expression plasmids carrying a modified target gene sequence, ready for transformation of a desired expression host. These exact methods were applied for the assembly of the expression plasmids for recombinant protein production or CRISPR applications in peer-reviewed and published articles [3,4]. From our experience, the presented advanced primer cloning approaches are very robust and reproducible. Still, quantification of the success rate is not easily possible, as the handling of the operator may have a significant impact. We routinely send two plasmid preparations per construct for sequencing, and almost always, both or at least one of the plasmids contains the correctly modified DNA. Also, since the plasmids are sequenced before further usage, it is very unlikely that a failed cloning approach jeopardizes further experiments.

Other outcomes, such as the yield of the purified, PCR linearized expression plasmid, or the amount of plasmid DNA from the final plasmid isolation, are potentially very dependent on the experience of the user, the kits, plasmids, and strains used. In our experience, a qualified person using the kits and reagents mentioned in this method will obtain 30 µl of ∼200 ng/µl of the linearized purified PCR product, whereas a beginner will potentially have concentrations <100 ng/µl. Similarly, an experienced user will manage to obtain 50 µl of 300 to 700 ng/µl of isolated plasmid DNA, whereas inexperienced users will end up with the same volume but plasmid concentrations of 200–300 ng/ µl.

Still, all these numbers don’t really influence the assembly process, as only small amounts of DNA are required, meaning that even inexperienced users will obtain the DNA needed for the transformation of the final expression host.

## Limitations

The cloning approach itself is robust, but some subjects should be considered. Firstly, despite their high fidelity, the polymerases used in the PCR are not errorless. Thus, the longer the DNA that is amplified for the cloning process, the higher the chance that the final plasmid contains unwanted mutations. Still, using typical expression plasmids of the size of ∼4000 bp in combination with the Phusion Polymerases, in theory, only ∼5% of the amplified DNA molecules will have one error after 30 cycles. Also, plasmids are sequenced before use, providing a sufficient solution to this limitation.

Secondly, it is worth considering the economic aspects of the approach. For us, the presented applications are well-suited because we have access to cost-effective primers with short delivery times, enabling efficient adaptation of our plasmids. We routinely order primers of up to 60 nt in length, as they are inexpensive. Primers longer than 60 nt become increasingly expensive and quickly reach a point at which commercially available synthetic double-stranded DNA fragments (e.g., gBlocks Gene Fragments from IDT or synthetic DNA fragments from Twist Bioscience) represent an economically attractive alternative, particularly for introducing longer or more complex DNA sequences.

Finally, it is worth considering handling capacity. We do not recommend performing more than 24 assemblies simultaneously, as this is typically the number of samples that fit in a single thermomixer and bench-top centrifuge and can thus be conveniently handled by one person. In addition, 24 assemblies will result in 48–96 plasmid preparations and sequencing reactions when following the method described here.

## Ethics statements

Not applicable

## CRediT author statement

LR wrote the first draft of the manuscript; LR, AKH, AMH, VH, FW tested and validated the approaches; AKH, AMH, VH, FW, AG edited the manuscript; AG and LR provided funding and infrastructure. All authors agreed on the final version of the manuscript.

## Declaration of competing interest

The authors declare that they have no known competing financial interests or personal relationships that could have appeared to influence the work reported in this paper.

## Data Availability

Data will be made available on request.
